# Exploring the early diagnostic value of MRI for type I stress fractures: a retrospective analysis based on imaging manifestations

**DOI:** 10.3389/fsurg.2025.1333714

**Published:** 2025-03-17

**Authors:** Hui Lu, Cailin Wang, Leilei Wang, Xuefeng Gao, Ruquan Li, Xiaofeng Jin, Jun Tang, Sen Guan

**Affiliations:** ^1^ Department of Pain, Suzhou Hospital of Integrated Traditional Chinese and Western Medicine, Suzhou, China; ^2^ Medical Research Center, The People’s Hospital of Suzhou New District, Suzhou, China; ^3^ Department of Radiology, Yinshanhu Hospital, Suzhou, China; ^4^ Department of Radiology, The 904th Hospital of the People’s Liberation Army (PLA) Joint Logistics Support Force, Suzhou, China

**Keywords:** stress fractures, magnetic resonance imaging, early diagnosis, military training, imaging manifestations

## Abstract

**Objective:**

To compare the positive rate of early diagnosis and the detection rate of fracture signs in Type I stress fractures using x-ray, CT, and MRI.

**Methods:**

A total of 56 patients with Type I stress fractures admitted to the 904st Hospital of the PLA Joint Logistics Support Force from January 2011 to June 2021 were included in the retrospective analysis, including 35 cases of tibial stress fractures (tibia group) and 21 cases of femoral stress fractures (femur group). The positive rate of early diagnosis and the detection rates of visible fracture lines, periosteal reaction, callus formation, surrounding soft tissue swelling, and marrow cavity signal changes were compared between x-ray, CT, and MRI.

**Results:**

**(**1) The positive rate of early diagnosis of MRI in the tibia and femur groups were significantly higher than those of x-ray and CT examinations, and the differences were statistically significant (*P* < 0.05). (2) In the tibia group, MRI had significantly higher detection rates than x-ray and CT examinations for visible fracture lines, periosteal reaction, surrounding soft tissue swelling, and marrow cavity signal changes, and the differences were statistically significant (*P* < 0.05). There was no significant difference in the detection rate of callus formation (*P* > 0.05). (3) In the femur group, MRI had significantly higher detection rates than x-ray and CT examinations for visible fracture lines, surrounding soft tissue swelling, and marrow cavity signal changes, and the differences were statistically significant (*P* < 0.05). There was no significant difference in the detection rates of periosteal reaction and callus formation (*P* > 0.05).

**Conclusion:**

Based on the definitely diagnostic advantages of MRI for signs such as visible fracture line, surrounding soft tissue swelling, and marrow cavity signal changes, it shows higher accuracy and application value in the early diagnosis of type Ⅰ stress fractures.

## Introduction

Stress fractures, as a frequent chronic injury in military training, pose a significant health challenge to military personnel, stemming from the long-term exposure of the skeletal system to non-physiological stresses (1–4). When muscles are pushed beyond their absorption capacity due to excessive use, the vibrations resulting from repeated impacts, which should normally be absorbed by the muscles, are directly transferred to the bones. This long-term, repeated direct or indirect trauma gradually weakens the bone structure, ultimately leading to small or localized fractures. This type of fracture is also known as “march fracture” due to its close association with prolonged marching or running training (5, 6).

Stress fractures commonly occur in weight-bearing or impact-prone areas such as the tibia, femur, fibula, second metatarsal, and radius, where bones are more susceptible to damage during high-intensity training. It is noteworthy that early-stage stress fractures often lack a typical history of trauma and may present as negative on x-ray examinations, which complicates the diagnostic process. Coupled with the intense training volume, strict management requirements, and potential constraints in medical resource allocation within military settings, cases of missed diagnosis, misdiagnosis, and treatment delays are not uncommon (7–9). Therefore, enhancing early diagnosis capabilities for stress fractures and strengthening preventive measures are crucial to reducing non-combat attrition within military units. By improving the diagnostic skills of medical personnel, strengthening health education and protective awareness among military personnel, and optimizing training plans, we can not only decrease the incidence of stress fractures but also further elevate the training quality and overall combat effectiveness of our military (10–14). These measures are not only vital for safeguarding the health of military personnel but also pivotal for enhancing our national defense capabilities.

X-ray examination, as a fundamental tool in orthopedic diagnosis, often struggles when dealing with early stress fractures. Due to the subtle and inconspicuous fracture lines in the initial stages of stress fractures, x-ray films often fail to capture these delicate signs of fracture (15). Typically, it is only after about 2 weeks, when bone and periosteal proliferation and sclerosis become evident, that x-ray films can more clearly reflect the presence of the fracture. However, this delayed visualization characteristic results in a relatively low detection rate of early stress fractures with x-ray films, making it difficult to meet the clinical needs for early diagnosis. CT scanning, as a more refined imaging technique, although to some extent improves the capture of detailed bone structure, still has limitations in displaying microdamage to the trabeculae of early stress fractures. Additionally, CT's insufficient sensitivity to changes in marrow cavity signals further complicates its use in diagnosing early stress fractures (16). Ultrasound diagnosis, as a non-invasive and real-time imaging technique, has also been applied in the diagnosis of stress fractures in recent years. It primarily monitors changes in the periosteum at the fracture site and blood flow perfusion to assist in diagnosis. However, ultrasound imaging has limitations in observing the comprehensive internal imaging of the cortical bone in early stages of fractures, making it difficult to fully meet the clinical demand for precise diagnosis (17). Meanwhile, Vibroarthrography, as an emerging diagnostic technique, has gradually been applied in the study of degenerative bone and joint diseases and fracture cracks in recent years. This technique measures the sounds and vibrations produced by the joint during movement, enabling early detection of internal degeneration and anatomical structure damage within the joint (18). However, despite its many potential advantages, Vibroarthrography faces numerous challenges in practical applications. The precise placement of sensors and the high requirements for noise reduction technology pose significant demands on researchers. Furthermore, anatomical variability and the lack of clear guidelines for sensor placement pose challenges to the promotion and application of Vibroarthrography. In contrast, MRI, as an advanced imaging technique, demonstrates exceptional performance in diagnosing stress fractures. It not only clearly displays changes within the marrow cavity, including trabeculae, microvessel ruptures, and changes in marrow water signals, but also captures early changes in cortical bone and periosteum. More importantly, MRI can also definitively show damage signals in the muscles, tendons, and ligaments surrounding the fracture, providing clinicians with more comprehensive and accurate diagnostic information. Due to its non-invasiveness, multi-angle imaging, multi-parameter capabilities, and high signal sensitivity, MRI is considered by some studies to be the gold standard (19) for diagnosing stress fractures and the best examination method for early diagnosis of stress fractures.

This article aims to comparatively analyze the imaging differences among x-ray, CT, and MRI in diagnosing type I stress fractures at various locations such as the tibia and femur. It analyzes the positive diagnostic rates of x-ray, CT, and MRI for fractures, as well as the detection rates of visible fracture lines, periosteal reaction, callus formation, surrounding soft tissue swelling, and marrow cavity signal changes. The objective is to ascertain the effectiveness and accuracy of MRI in diagnosing type I stress fractures. Additionally, the limitations of this clinical study are discussed, and recommendations for the application and promotion of MRI technology in diagnosing type I stress fractures are proposed.

## Materials and methods

### Clinical data

A total of 105 patients with Type I stress fractures (20, 21) were selected from the clinical records of the 904th Hospital of the People's Liberation Army (PLA)Joint Logistics Support Force, from January 2011 to June 2021. Among them, 56 patients with complete follow-up data were included in the analysis, including 35 cases of tibial stress fractures (tibia group) and 21 cases of femoral stress fractures (femur group).

This study was approved by the Ethics Committee of the 904th Hospital of the People's Liberation Army (PLA) Joint Logistics Support Force, and informed consent was obtained from the patients and their family members.

### Inclusion criteria

(1) No history of significant trauma; (2) History of high-intensity military training for at least 1 week; (3) Patients presented with localized pain, with or without swelling, tenderness and percussion tenderness (+), exacerbation after activity, and alleviates after rest; (4) Signed informed consent form.

### Exclusion criteria

(1) Patients with underlying diseases such as lymphoma, multiple myeloma, hyperparathyroidism, rheumatoid arthritis, which may cause osteoporosis; (2) Patients take the following drugs for a long time, such as glucocorticoids, immunosuppressants, or other medications; (3) Patients with mental disorders who cannot cooperate with examinations and treatment.

### Imaging methods

X-ray imaging was performed using the New Eastern 1000D medical x-ray radiography system. CT scans were performed using the Aqilion whole-body CT machine. Routine scans were conducted at the tenderness area and its superior and inferior layers, with a layer thickness and interlayer spacing of 3 mm. MRI scans were performed using the Brivo MR355 1.5T superconducting MR imaging system. Multi directional imaging through sagittal, coronal, and transverse views, including spin echo (SE) sequences, fast spin echo (FSE) sequences, T1-weighted imaging (TR 300 TE 102), T2-weighted imaging (TR 3046 TE 42), sagittal fat-suppressed proton density (TE 36 TR 2000). The layer thickness was 5 mm and the interlayer spacing is1–2 mm.

### Image interpretation and evaluation criteria

All collected imaging data were evaluated by two experienced radiologists. The diagnostic positive rates of x-ray, CT, and MRI for different locations of stress fractures were compared. The detection rates of visible fracture lines, periosteal reaction, callus formation, surrounding soft tissue swelling, and marrow cavity signal changes in stress fractures were analyzed.

### Statistical analysis

Statistical analysis was performed using SPSS 16.0 software. Continuous data were expressed as mean ± standard deviation (*X* *±* *S)*, and independent sample *t*-tests were used. Categorical data were expressed as percentages (*%*), and chi-square tests were used. A significance level of *P* < 0.05 indicated statistical significance.

## Results

### Comparison of general information between two groups of patients

The tibia group consisted of 35 male cases and 0 female cases, with an average age of (20.63 ± 2.90) years, a mean duration of illness of (14.40 ± 27.94) weeks, 18 cases on the left side, 12 cases on the right side, and 5 cases with bilateral involvement. The femur group consisted of 20 male cases and 1 female case, with an average age of (19.95 ± 5.64) years, a mean duration of illness of (11.30 ± 22.23) weeks, 13 cases on the left side, 8 cases on the right side, and 0 cases with bilateral involvement. There were no statistically significant differences in gender, age, duration of illness, or injury site between the two groups (*P* > 0.05).

### Comparison of the positive rates of early diagnosis for tibial and femoral stress fractures among the three imaging methods

Comparative analysis of the three imaging methods showed that MRI had significantly higher early diagnosis positive rates and accuracy than x-ray and CT examinations in both the tibia and femur groups, with statistically significant differences (*P* *<* 0.05) (Table 1). The MRI manifestations of stress fractures were as follows: the affected area showed large patchy high signal intensity on T1-weighted imaging (T1WI) and T2-weighted imaging (T2WI), with blurred boundaries. In the coronal or sagittal position, horizontal or oblique bands of low T1WI and low T2WI signals can be seen penetrating the bone cortex. Surrounding soft tissue swelling was more clearly displayed on the fat-suppressed sequences of T2WI. However, periosteal reaction showed low signal intensity on both T1WI and T2WI, similar to the signal intensity of the cortical bone.

**Table 1:** Comparison of early diagnosis positive rates for tibial and femoral stress fractures among the three imaging modalities (*n, %*)**.**

Site	Imaging modality			*P*_1_	*χ^2^*	*P*_2_	*χ^2^*	*P*_3_	*χ^2^*
	x-ray	CT	MRI						
Tibia (*n* = 35)	9 (25.71)	21 (60.00)	35 (100.00)	0.004	8.400	0.000	41.364	0.000	17.500
Femur (*n* = 21)	10 (47.62)	15 (71.43)	21 (100.00)	0.116	2.471	0.000	14.903	0.008	7.000

P1 refers to the comparison of detection rates between the x-ray group and the CT group. P2 refers to the comparison of detection rates between the x-ray group and the MRI group. P3 refers to the comparison of detection rates between the CT group and the MRI group.

### Comparison of imaging manifestations of type I stress fracture of tibia

**Figure 1:**
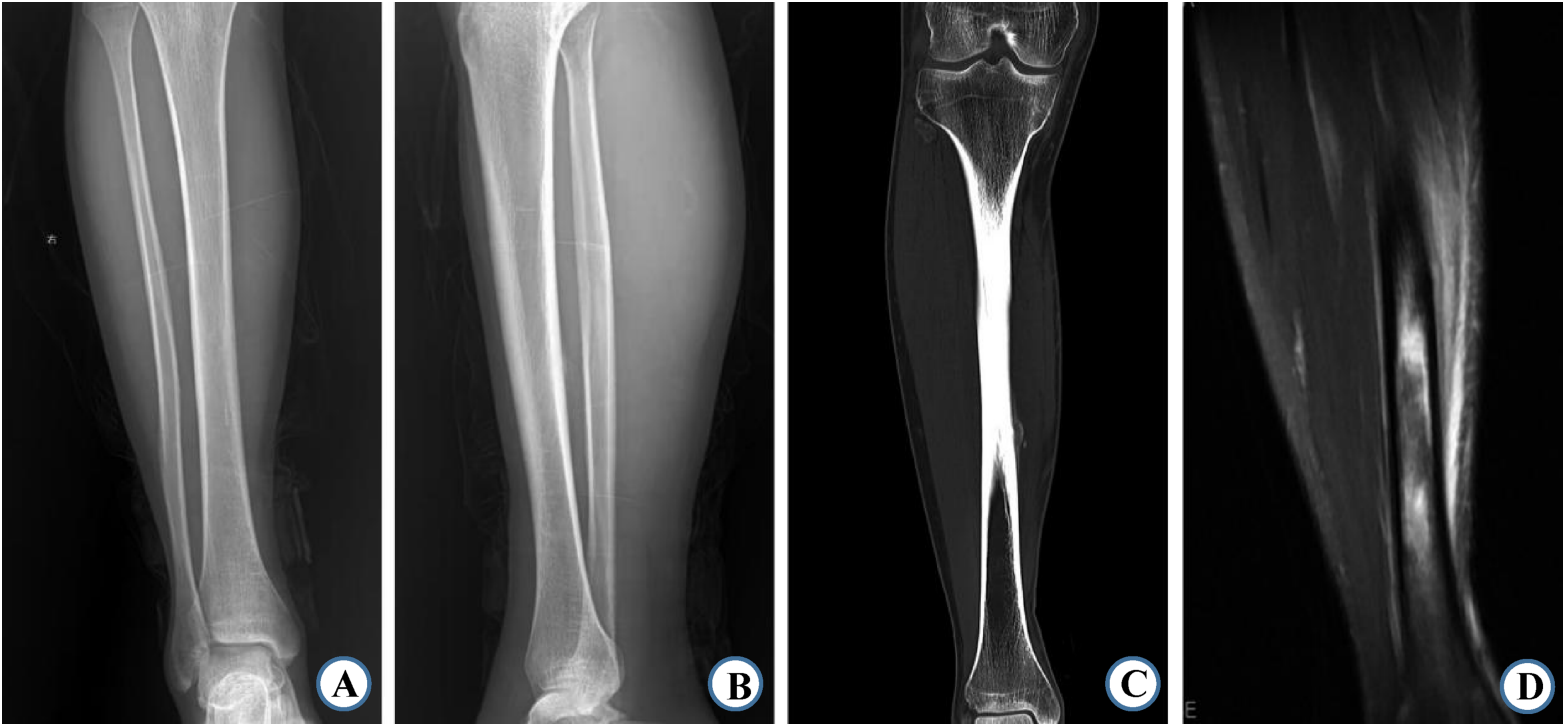
A 19-year-old male with a right tibial Type I stress fracture. **(A,B)** No significant abnormality observed on x-ray in the anteroposterior and lateral views. **(C)** CT scan shows localized thickening of the cortical bone in the middle segment of the right tibia. **(D)** MRI reveals a linear low-signal intensity area in the middle segment of the right tibia, surrounding patchy high-signal intensity areas on T2-weighted fast spin echo (T2FSE), and inner soft tissue edema.

In the tibia group, MRI examination showed significantly higher detection rates than x-ray and CT examinations for visible fracture lines, periosteal reaction, surrounding soft tissue swelling, and marrow cavity signal changes (*P* < 0.05) (Table 2; Figure 1). However, there was no significant difference in the detection rate of callus formation among the three imaging methods (*P* > 0.05).

**Table 2:** Comparison of detection rates for imaging findings in tibial type Ⅰ stress fractures (*n, %*)**.**

Imaging findings	x-ray	CT	MRI	*P*_1_	*χ^2^*	*P*_2_	*χ^2^*	*P*_3_	*χ^2^*
Visible fracture line	9 (25.71)	10 (28.57)	26 (74.29)	0.788	0.072	0.000	16.514	0.000	14.641
Periosteal reaction	0 (0.00)	6 (17.14)	23 (65.71)	0.010	6.562	0.000	34.255	0.000	17.014
Callus formation	8 (22.86)	8 (22.86)	9 (25.71)	1.000	0.000	0.780	0.078	0.780	0.078
Surrounding soft tissue swelling	2 (5.71)	5 (14.29)	13 (37.14)	0.232	1.429	0.001	10.267	0.029	4.786
Marrow cavity signal changes	0 (0.00)	7 (20.00)	35 (100.00)	0.005	7.778	0.000^a^	66.057^a^	0.000	46.667

P1 refers to the comparison of detection rates between the x-ray group and the CT group. P2 refers to the comparison of detection rates between the x-ray group and the MRI group. P3 refers to the comparison of detection rates between the CT group and the MRI group.

^a^ Indicates the result obtained using the continuity-corrected chi-square test.

### Comparison of imaging manifestations of type I stress fracture of femur

**Figure 2:**
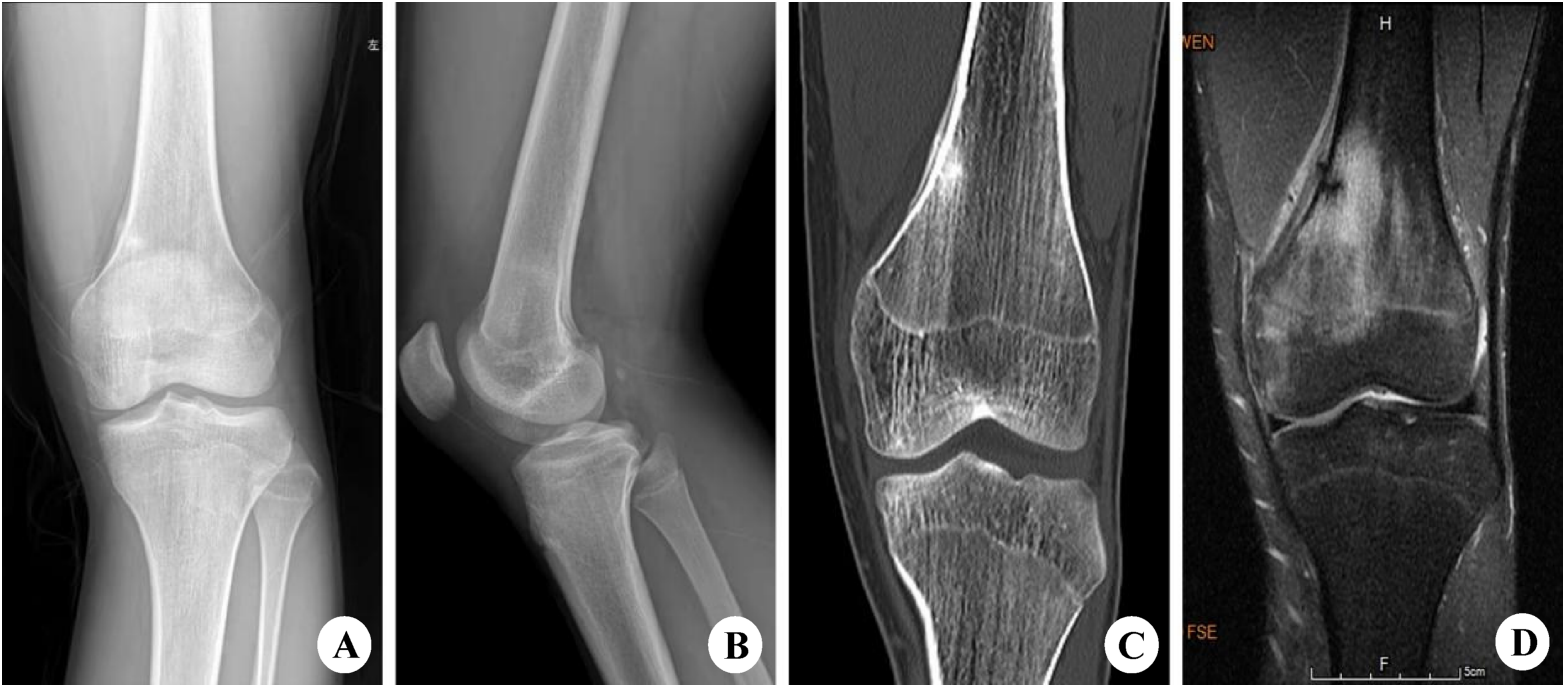
A 20-year-old male with a left femoral Type I stress fracture. **(A,B)** On the anteroposterior and lateral x-ray views, increased cortical density is observed in the distal region of the left femur. **(C)** CT scan reveals discontinuity of the cortical bone with strip-like high-density shadows along the edges in the distal region of the left femur. **(D)** MRI shows signs of fracture line at the distal end of the left femur, accompanied by large areas of long T1 and long T2 signal intensity in the bone marrow cavity.

MRI examination in the femur group showed significantly higher detection rates than x-ray and CT examinations for visible fracture lines, surrounding soft tissue swelling, and marrow cavity signal changes (*P* < 0.05). However, there was no significant difference in the detection rates of periosteal reaction and callus formation among the three imaging methods (*P* > 0.05) (Table 3; Figure 2).

**Table 3:** Comparison of detection rates for imaging findings in femoral type Ⅰ stress fractures (*n*, *%*)**.**

Imaging findings	x-ray	CT	MRI	*P*_1_	*χ^2^*	*P*_2_	*χ^2^*	*P*_3_	*χ^2^*
Visible fracture line	10 (47.62)	14 (66.67)	20 (95.24)	0.212	1.556	0.001	11.667	0.018	5.559
Periosteal reaction	1 (4.76)	4 (19.05)	3 (14.29)	0.341^a^	0.908^a^	0.599^a^	0.276^a^	1.000^a^	0.000^a^
Callus formation	4 (19.05)	4 (19.05)	3 (14.29)	1.000^a^	0.000^a^	1.000^a^	0.000^a^	1.000^a^	0.000^a^
Surrounding soft tissue swelling	0 (0.00)	0 (0.00)	8 (38.10)	–	–	0.002	9.882	0.002	9.882
Marrow cavity signal changes	0 (0.00)	2 (9.52)	21 (100.00)	0.469^a^	0.525^a^	0.000^a^	38.095^a^	0.000^a^	31.140^a^

P1 refers to the comparison of detection rates between the x-ray group and the CT group. P2 refers to the comparison of detection rates between the x-ray group and the MRI group. P3 refers to the comparison of detection rates between the CT group and the MRI group.

^a^ Indicates the result obtained using the continuity-corrected chi-square test.

## Discussion

Repetitive non-physiological stress below the threshold of bone strength on weight-bearing areas is the fundamental cause of stress fractures (8, 22). Initially, patients only experience localized pain after activity, which significantly improves with rest. However, the bone already undergoes microfractures of trabeculae, microvascular damage, and bone marrow edema (23), which are not easily detectable by x-ray or CT scans. At this stage, MRI demonstrates its unique advantages (24–29). MRI examination can not only detect early signs of microfractures, microvascular damage, and bone marrow signal changes but also reveal changes in surrounding soft tissues such as periosteum, muscles, tendons, and ligaments. Therefore, some researchers consider MRI as the “gold standard” for early diagnosis of stress fractures (30–32). Ziesler et al. (33) suggest that pain and clinical manifestations in the hip or inguinal region of athletes should be taken seriously, and a definitive diagnosis relies on MRI examination to determine the location and stability of the fracture, guiding conservative or surgical treatment. Harrasser et al. (34) state that within the first 4–6 weeks, x-ray examinations for stress fractures are often negative, and early MRI should be employed for exclusion. Song et al. (35) emphasize that medical history and physical examination are the basis for diagnosing bone stress injuries, and magnetic resonance imaging helps confirm the diagnosis and classify the severity. Consequently, a substantial body of literature confirms the definite application value of MRI in the early diagnosis of stress fractures, effectively reducing misdiagnosis, missed diagnosis, and treatment delays (36, 37).

Certainly, MRI also has certain drawbacks. Factors such as the initial investment in equipment, maintenance and repair costs, and the cost per examination contribute to the high expense of MRI, which can influence clinical decision-making by doctors and patients. In clinical practice, we will assess the cost-benefit ratio of MRI in diagnosing stress fractures. Considering its high sensitivity and specificity, MRI can lead to earlier and more accurate identification of stress fractures, potentially reducing morbidity and overall care costs. Ultimately, our goal is to inform clinical practice and policy decision-making by demonstrating the value of MRI as a cost-effective diagnostic tool for stress fractures. Notably, the latest research introduces a new, cheaper technology known as Vibroarthrography. By utilizing EEMD-DFA algorithms, reducing classifier inputs through ANOVA, and then classifying using artificial neural networks (ANN), a classification accuracy of nearly 93% was achieved. For the multilayer perceptron network, a sensitivity of 0.93, a specificity of 0.93, and an AUC of 0.942 were obtained (38). These results are encouraging, and if this technology is promoted for early screening of stress fractures in the military, with further validation of its accuracy through the collection of more samples, it could serve as a better alternative to MRI (39).

In this study, while confirming the early diagnostic value and accuracy of MRI in tibial and femoral stress fractures, we observed certain differences in the imaging findings between tibial and femoral stress fractures. We further conducted a comparative analysis of the imaging findings in tibial and femoral stress fractures. CT showed a significantly higher detection rate of “visible fracture lines” in femoral stress fractures compared to tibial stress fractures (*P* = 0.005, *χ^2^* = 7.778). MRI also exhibited a significantly higher detection rate of “visible fracture lines” in femoral stress fractures compared to tibial stress fractures (*P* = 0.047, *χ^2^* = 3.928), whereas MRI had a significantly lower detection rate of “periosteal reaction” in femoral stress fractures compared to tibial stress fractures (*P* = 0.000, *χ^2^* = 13.957). This indicates that stress fractures of the femur are more likely to manifest as cortical bone fractures with less prominent periosteal proliferation, whereas stress fractures of the tibia exhibit more significant periosteal proliferation. We hypothesize that the following factors may contribute to this difference: (1) Bone structure and mechanical properties: As one of the longest bones in the human body, the femur has a relatively thick cortical bone that can withstand considerable pressure and load. Therefore, under prolonged repeated stress, the femur is more prone to cortical bone fractures. In contrast, although the cortical bone of the tibia also possesses certain strength, its structural characteristics may make it more susceptible to eliciting a periosteal response when subjected to repeated stress, leading to periosteal proliferation. (2) Muscle attachment and stress distribution: The muscle groups around the femur are relatively stronger, providing more significant support and protection to the femur. However, this may also result in the femur bearing excessive stress in some cases, thereby causing cortical bone fractures. The muscles around the tibia are relatively fewer, and its stress distribution may be more complex. This complex stress distribution may make the tibia more prone to periosteal proliferation when subjected to repeated stress. (3) Biomechanical factors: Different exercise modes and activity levels may also influence the manifestation of stress fractures in the femur and tibia. For example, certain exercises may involve more force on the femur, while others may primarily affect the tibia. Additionally, factors such as an individual's weight, height, and gait may also impact the stress distribution in the femur and tibia, thereby affecting the manifestation of stress fractures. (4) Nutritional and metabolic factors: The health of bones is closely related to an individual's nutritional intake and metabolic level. Issues such as malnutrition or metabolic abnormalities may affect bone strength and toughness, thereby increasing the risk of stress fractures. It is worth noting that although nutritional and metabolic factors may influence the manifestation of stress fractures in both the femur and tibia, they may do so through different mechanisms.

Additionally, this study has certain limitations. It focused only on the imaging findings of patients with stress fractures and lacked further correlation with clinical factors, treatment plans, prognosis, and pain relief, which should be addressed in our future research. And, we must point out that the limited sample size potentially impacts the generality and robustness of our study results. To address this limitation, we plan to undertake further research in several directions. Firstly, we intend to expand the scope of our study by including a larger and more diverse group of participants. This will help to ensure that our findings are more representative of the broader population and increase the statistical power of our analysis. Secondly, we are exploring the possibility of collaborating with other research institutions or clinical centers to conduct a multi-center study. Such collaboration would not only allow us to access a larger pool of participants but also provide a more comprehensive view of the condition across different settings and patient populations.

## Conclusion

In summary, compared to x-ray and CT examinations, MRI demonstrates clear diagnostic advantages in visible fracture lines, surrounding soft tissue swelling, and marrow cavity signal changes, making it a more accurate and valuable tool for the early diagnosis of Type I stress fractures in various locations.

## Data Availability

The raw data supporting the conclusions of this article will be made available by the authors, without undue reservation.
